# Dominant inheritance of retinal ganglion cell resistance to optic nerve crush in mice

**DOI:** 10.1186/1471-2202-8-19

**Published:** 2007-03-05

**Authors:** Yan Li, Sheila J Semaan, Cassandra L Schlamp, Robert W Nickells

**Affiliations:** 1 Department of Ophthalmology and Visual Sciences, University of Wisconsin, Madison, Wisconsin, USA

## Abstract

**Background:**

Several neurodegenerative diseases are influenced by complex genetics that affect an individual's susceptibility, disease severity, and rate of progression. One such disease is glaucoma, a chronic neurodegenerative condition of the eye that targets and stimulates apoptosis of CNS neurons called retinal ganglion cells. Since ganglion cell death is intrinsic, it is reasonable that the genes that control this process may contribute to the complex genetics that affect ganglion cell susceptibility to disease. To determine if genetic background influences susceptibility to optic nerve damage, leading to ganglion cell death, we performed optic nerve crush on 15 different inbred lines of mice and measured ganglion cell loss. Resistant and susceptible strains were used in a reciprocal breeding strategy to examine the inheritance pattern of the resistance phenotype. Because earlier studies had implicated *Bax *as a susceptibility allele for ganglion cell death in the chronic neurodegenerative disease glaucoma, we conducted allelic segregation analysis and mRNA quantification to assess this gene as a candidate for the cell death phenotype.

**Results:**

Inbred lines showed varying levels of susceptibility to optic nerve crush. DBA/2J mice were most resistant and BALB/cByJ mice were most susceptible. F1 mice from these lines inherited the DBA/2J phenotype, while N2 backcross mice exhibited the BALB/cByJ phenotype. F2 mice exhibited an intermediate phenotype. A Wright Formula calculation suggested as few as 2 dominant loci were linked to the resistance phenotype, which was corroborated by a Punnett Square analysis of the distribution of the mean phenotype in each cross. The levels of latent *Bax *mRNA were the same in both lines, and *Bax *alleles did not segregate with phenotype in N2 and F2 mice.

**Conclusion:**

Inbred mice show different levels of resistance to optic nerve crush. The resistance phenotype is heritable in a dominant fashion involving relatively few loci. *Bax *was excluded as a candidate gene for this phenotype.

## Background

The influence of complex genetics on neurodegenerative diseases such as Alzheimer's Disease, Parkinson's Disease, and Multiple Sclerosis, is evidenced by the consideration that family history is a significant risk factor for candidate patients [1-3]. Although interacting loci may influence a variety of different factors, one common element is the process of neuronal cell death, which predominantly occurs by apoptosis [4,5]. Consequently, consideration of the genes that affect susceptibility to disease should include those that affect both the response(s) of neurons to damaging stimuli and the process of cell death itself. Several studies, using screening strategies of inbred mouse lines, have reinforced the concept that genetic background has an influence on the level of susceptibility to neuronal damage [6-9]. At least 3 loci have been mapped, for example, that affect neuronal susceptibility to the Parkinsonian neurotoxin 1-methyl-4-phenyl-1,2,3,6-tetrahydropyridine (MPTP) [10,11].

Primary open angle glaucoma is also a multifactorial neurodegenerative disease with a strong familial component [12-14]. This disease affects an estimated 60 million individuals worldwide [15] and is characterized by the progressive apoptotic death of retinal ganglion cells and the degeneration of the optic nerve [16-18]. All forms of glaucoma are associated with critical elevations in intraocular pressure (IOP), but humans exhibit a wide range of susceptibility to this stimulus. Although most glaucoma patients have a higher than average IOP, the majority of ocular hypertensives will never develop the disease [19,20]. Conversely, there is a significant percentage of glaucoma sufferers who have normal or below normal IOP [17]. In the majority of people with glaucoma, however, lowering IOP regardless of the starting level, has a beneficial effect in slowing the progression of the disease. This clinical history of disease progression and response to treatment has led to the concept that there is variable susceptibility to the damaging effects of eye pressure [20-22].

One genetically regulated component that could influence ganglion cell susceptibility is the cell death process itself. In response to a majority of cell death stimuli, retinal ganglion cell death in mice is restricted to *Bax*-dependent, intrinsic apoptosis, a pathway common to many neurons of the CNS. This pathway is activated during development [23], after acute optic nerve lesion [24], or in chronic spontaneous glaucoma [25]. Since apoptosis is an intrinsic genetic process, differences in alleles of genes that affect cell death could influence ganglion cell susceptibility to a damaging stimulus. Reducing *Bax *gene dosage, for example, reduces the level of latent *Bax *mRNA and can completely abrogate ganglion cell death after optic nerve crush or in glaucoma [25]. To explore the possibility that genetic background could affect neuronal cell death, we first investigated if different strains of inbred mice exhibited different levels of ganglion cell loss in response to a standardized lesion of the optic nerve (mechanical crush). Here, we report that inbred mice have different susceptibilities to optic nerve crush, and that the resistant phenotype can be inherited in a relatively simple Mendelian fashion. Although *Bax *plays a critical role in regulating ganglion cell susceptibility, we excluded it as a candidate gene for this trait. Identification of the gene(s) that affect the susceptibility of retinal ganglion cells to damaging stimuli could help elucidate part of the genetic complexity of glaucoma and other neurodegenerative diseases.

## Results

### Inbred mice exhibit quantitative differences in ganglion cell loss after optic nerve crush

**Figure 1:**
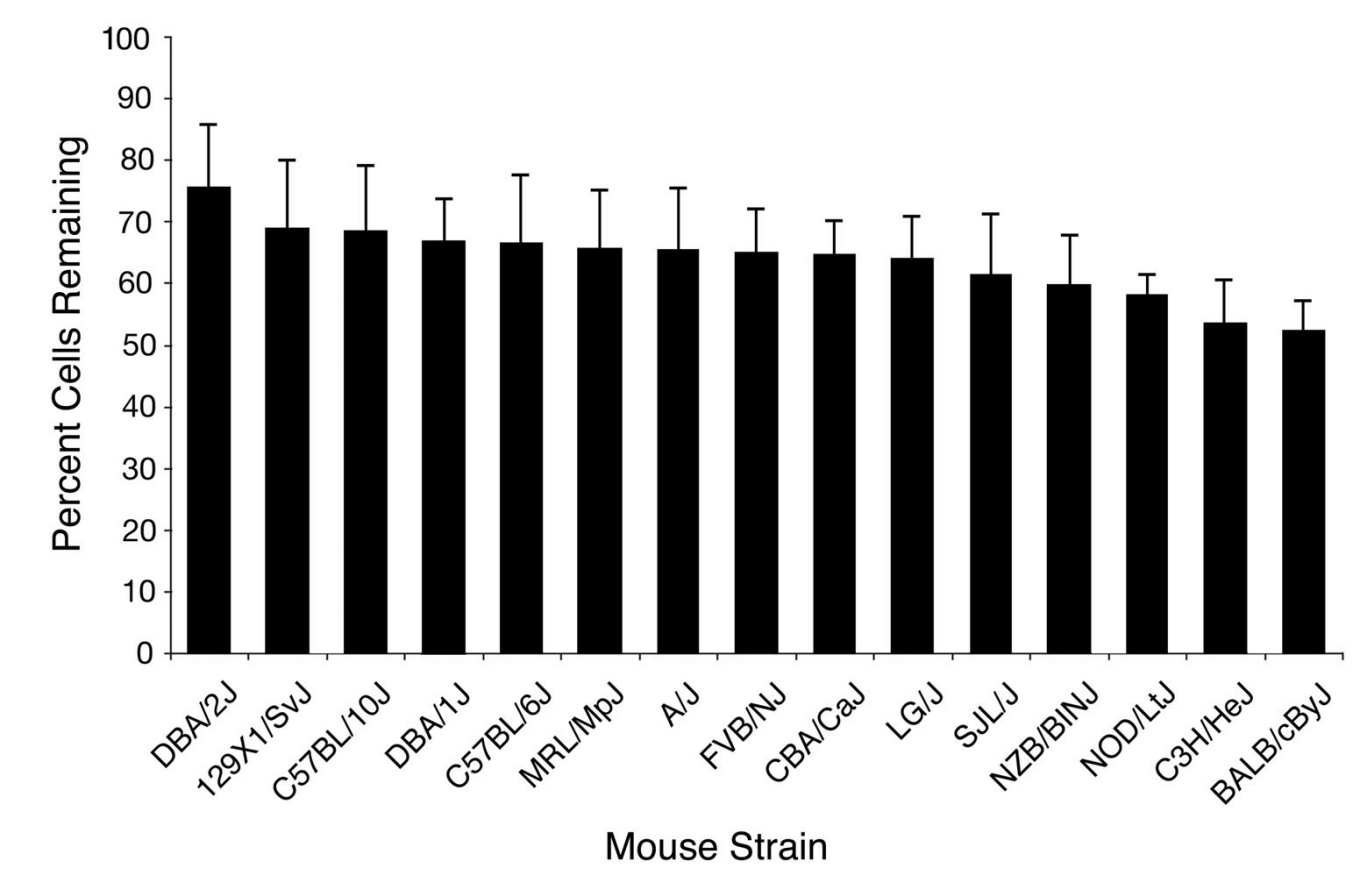
**Inbred mice exhibit different sensitivity to optic nerve crush**. Histographs showing the percentage of cells remaining in the retinal ganglion cell layer 2 weeks after optic nerve crush. The data shown is the mean (± SD) of 10 mice from each strain and reflects the percentage of cells present in the experimental retina relative to the fellow control eye.

**Figure 2:**
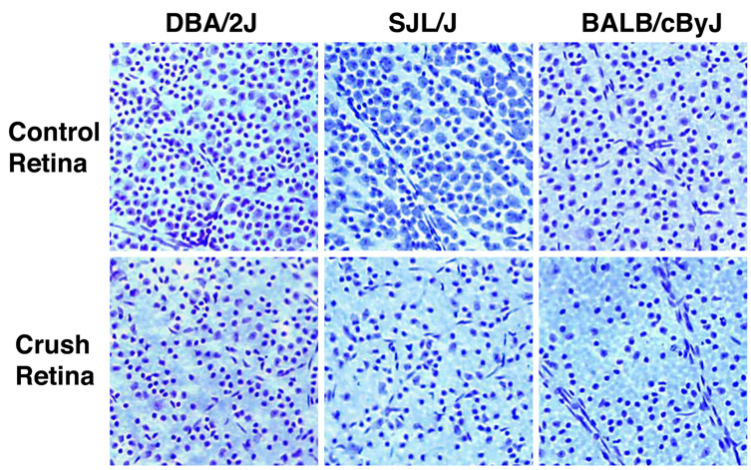
**Nissl-stained ganglion cell layers from control and experimental eyes**. Each panel is representative of the mid-central superior retina of the inbred lines shown. Two weeks after optic nerve crush, the retinas exhibit a loss of cells with larger somas and nuclei with prominent nucleoli, features characteristic of retinal ganglion cells. DBA/2J, SJL/J, and BALB/cByJ mice exhibit resistant, moderately resistant, and susceptible phenotypes to optic nerve crush, respectively. Magnification 200×.

Retinal ganglion cell loss after optic nerve crush occurs over a period of 3 weeks, with the most rapid period of loss occurring between the first and second weeks [26]. We chose to examine the period of 2 weeks after optic nerve crush in order to assess the process of cell death while still at an intermediate stage (see Methods section). A bar graph of the mean (± SD) of cells remaining in the crushed retina for 10 mice in 15 different lines is shown in Figure 1. Representative micrographs of control and crushed Nissl-stained retinas from mice at each end and the middle of this distribution are shown in Figure 2. Mice exhibited a range of cell loss after 2 weeks from 26.3% to 47.4% of the total neurons in the ganglion cell layer. A matrix of *P *values comparing each of the means against each other (*t*-tests) is shown in Table 1. Both sets of inbred lines with similar genetic backgrounds (DBA and C57BL mice) had comparable amounts of cell loss based on statistical testing. There were no significant correlations between coat color and susceptibility or known neurosensory defects and susceptibility (data not shown).

### Ganglion cell prevalence in DBA/2J and BALB/cByJ mice

From the initial screen of 15 inbred lines, DBA/2J mice were selected as the most resistant, and BALB/cByJ mice were selected as the most susceptible. A potential caveat in this study, however, is that cell loss was estimated as a percentage of the total neurons present in the ganglion cell layer. Since ganglion cells are not the only neurons present in this layer, our analysis of cell loss would only be accurate if ganglion cells comprised a similar percentage of the neurons in each strain. To assess this, we calculated the total number of neurons in the ganglion cell layers of DBA/2J and BALB/cByJ mice by counting cells in Nissl-stained whole mounts of untreated retinas. We also estimated the total number of ganglion cells in each strain by counting axons in sections of the optic nerves. Results of this analysis are shown in Table 2. Although DBA/2J mice have more total neurons in their ganglion cell layer they also have a proportionately higher number of ganglion cells, resulting in the same percentage of ganglion cells in both strains (~ 60%).

### Dominant inheritance of ganglion cell resistance to crush

**Figure 3:**
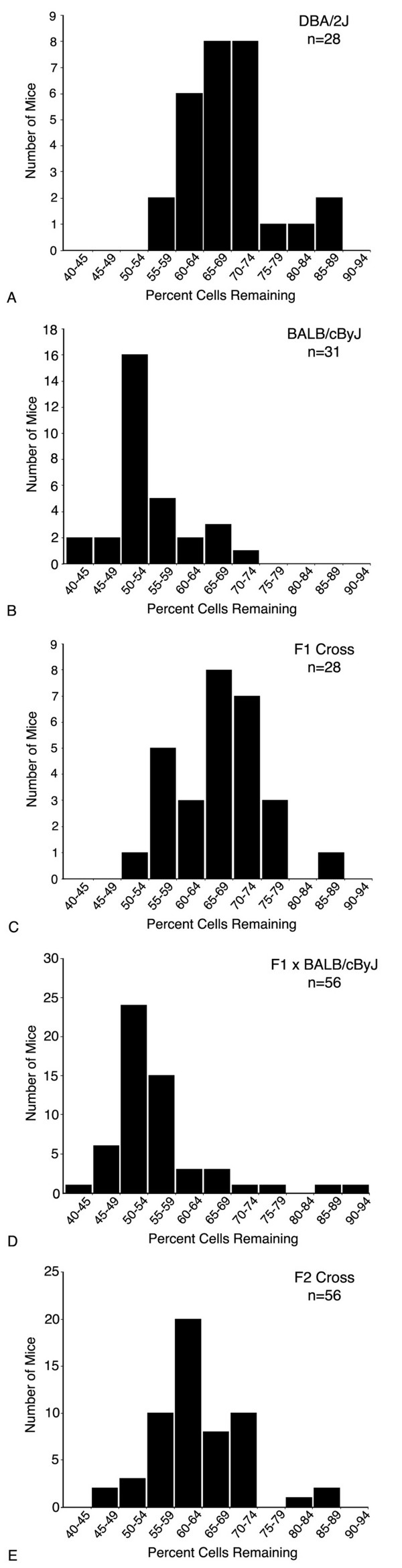
**Resistance to optic nerve crush is heritable**. Histographs showing the distribution of cell loss in 5 different populations of mice. (A) DBA/2J parental strain. (B) BALB/cByJ parental strain. (C) F1 generation of mice from DBA/2J and BALB/cByJ parents. These offspring inherit a level of resistance comparable to the DBA/2J parental strain. (D) N2 backcross generation of mice (F1 mice crossed with BALB/cByJ mice). These mice exhibit a shift toward the BALB/cByJ pattern of lowered resistance to optic nerve crush. (E) F2 generation of mice (F1 intercross). These mice exhibit an intermediate pattern of resistance.

To confirm these as resistant and susceptible strains, we performed optic nerve crush on increased numbers of mice from each strain (28 DBA/2J and 31 BALB/cByJ mice). Consistent with the initial screen, DBA/2J mice exhibited significantly less cell loss than BALB/cByJ mice (Figures 3A and 3B, *P *= 3.8 × 10^-8^, *t *test). To determine whether the susceptibility to optic nerve crush was genetically controlled, crosses were performed between the two strains. F1 mice (n = 28) inherited the optic nerve crush-resistance phenotype exhibited by DBA/2J mice (Figure 3C, *P *= 0.178 vs DBA/2J and *P *= 0.001 vs BALB/cByJ mice). N2 mice (n = 55), exhibited a shift toward a more susceptible phenotype (Figure 3D, *P *= 2.2 × 10^-6 ^vs DBA/2J and *P *= 0.15 vs BALB/cByJ). F2 mice (n = 56) yielded an intermediate phenotype after optic nerve crush with a bias toward the resistance phenotype (Figure 3E, *P *= 0.017 vs DBA/2J and *P *= 0.006 vs BALB/cByJ). By assigning a mean value for cell loss for the resistant and susceptible phenotypes, cell loss in each of the crosses was accurately predicted by the expected distribution of 2 dominant alleles using a Punnett Square method of analysis (data not shown).

A statistical estimation of the number of affecting loci was made using a modified Wright Formula, which takes into account the means and variances of the F1 mice and the recurrent backcross parental strain [27,28]. This formula yielded a value of 1.3, suggesting that only a few genes were contributing to the inheritance pattern of this phenotype, consistent with the Punnett Square analysis.

### Elimination of Bax as a contributing allele to the resistance phenotype

The proapoptotic gene *Bax *is essential for ganglion cell death after optic nerve crush [24] and in chronic spontaneous glaucoma [25]. Reduced *Bax *expression in mice heterozygous for a *Bax *knock-out allele has varying effects on neurons, depending on genetic background [25,29], an effect that appears to be related to the level of latent *Bax *mRNA from the endogenous wild type gene (S. Semaan and R. Nickells, unpublished observation) [30]. *Bax*^+/- ^DBA/2J mice are completely resistant to optic nerve crush [25] and have relatively low levels of neuronal *Bax *expression. Interestingly, DBA/2J mice were the most resistant to the same crush protocol of all the inbred mice examined.

**Figure 4:**
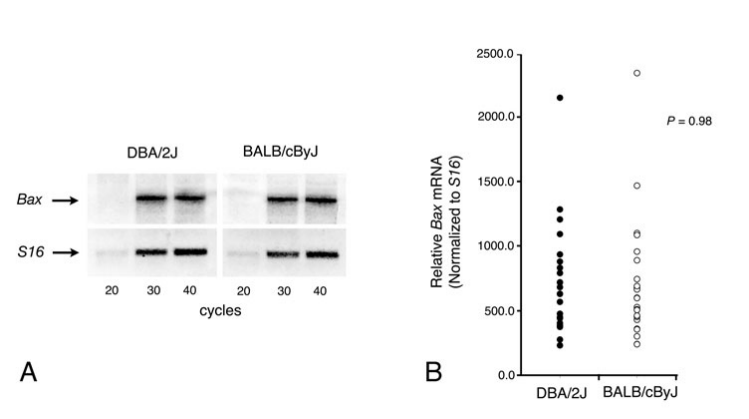
**Latent *Bax *mRNA levels are similar in the CNS of DBA/2J and BALB/cByJ mice**. (A) An ethidium bromide-stained gel (reverse image) showing the RT-PCR products for *Bax *and *S16 *mRNAs at increasing numbers of cycles. *Bax *mRNA appears equally abundant in both strains of mice. This observation was confirmed with RNase Protection Assays (B). The level of *Bax *message was normalized to the level of *S16 *and this value was plotted for each mouse examined. No statistical difference was observed in the *Bax *mRNA abundance between the two strains (*P *= 0.98, *t*-test).

**Figure 5:**
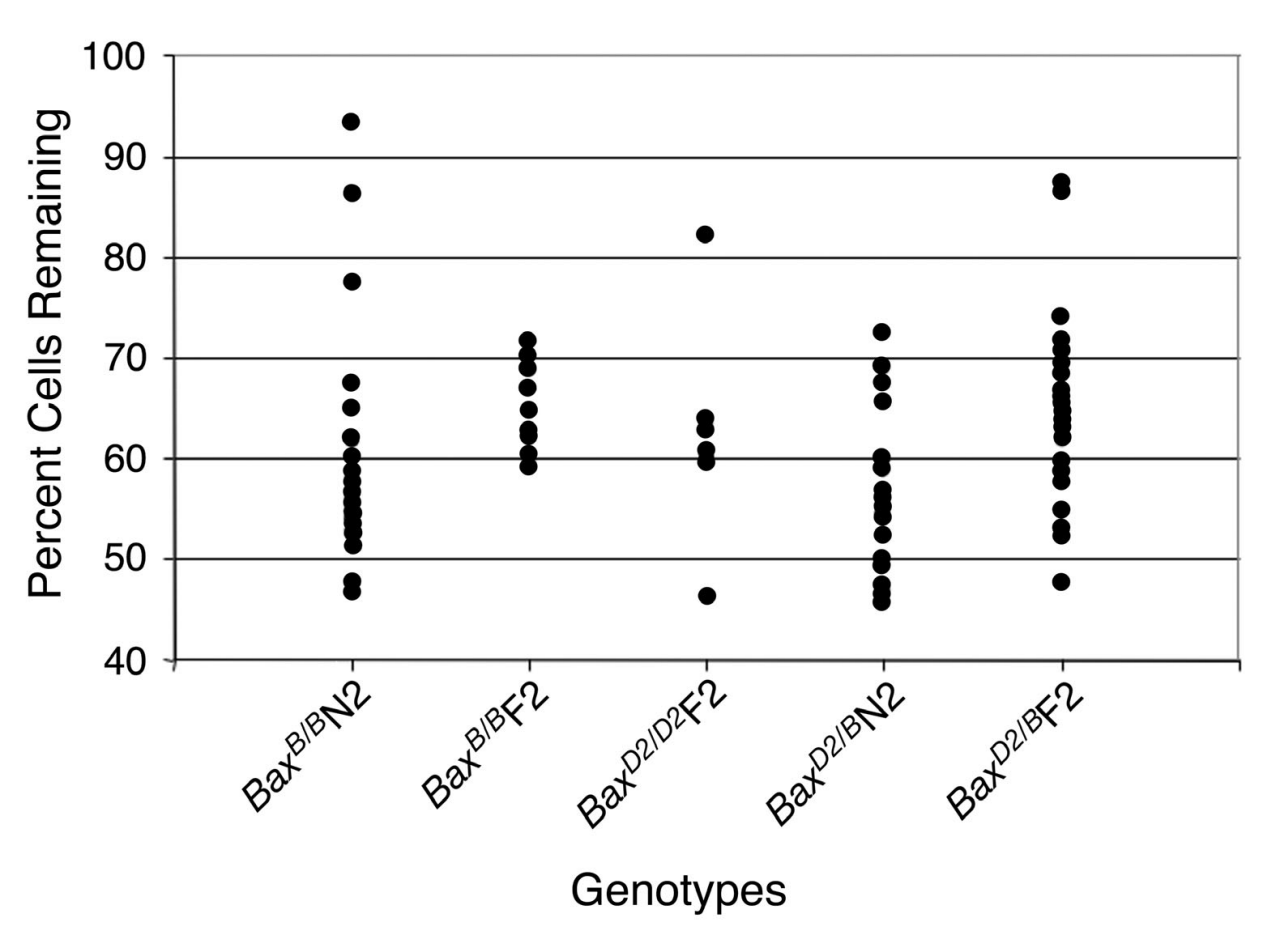
**Scatter plot showing the distribution of the percent cells remaining as a function of *Bax *allele and breeding population**. Mice from the N2 backcross and F2 populations were genotyped at position 23.0 on chromosome 7, near the location of the *Bax *allele. Each group of points represents individual animals carrying either a contributing allele from a BALB/cByJ parent (B) or a DBA/2J parent (D2). ANOVA analysis indicated no significant association between the resistance phenotype and the segregation of the *Bax *allele, excluding this gene as the principal contributor of this phenotype.

To examine *Bax *as a candidate gene for the resistant phenotype, we first quantified *Bax *mRNA levels in the brains of DBA/2J and BALB/cByJ mice using RNase Protection Assays (RPA). Both lines express a BAX protein with the same predicted amino acid sequence (data not shown), so if *Bax *was associated with the resistance phenotype, we predicted that BALB/cByJ mice would have higher levels of latent neuronal *Bax *mRNA. Quantitative examination of *Bax *mRNA levels in BALB/cByJ and DBA/2J mice showed no statistical difference between the two lines (*P *= 0.49, Figure 4). In a second experiment, we mapped the segregation of DBA/2J (D2) and BALB/cByJ (B) *Bax *alleles in N2 and F2 mice used in this study. A scatter plot of phenotype as a function of *Bax *genotype is shown in Figure 5. Evaluation of these data was made comparing mice with the *Bax*^B/B ^background against *Bax*^D2/B ^and *Bax*^D2/D2 ^mice (animals with the D2 allele were either pooled together as one group or compared as separate groups). None of the 4 possible comparisons yielded a significant association between the *Bax *genotype and the resistance phenotype (ANOVA, *P *= 0.150 for *Bax*^B/B ^vs. *Bax*^D2/B^and *Bax*^D2/D2 ^N2 and F2 mice combined, n = 106; *P *= 0.356 for *Bax*^B/B ^vs. *Bax*^D2/B ^vs. *Bax*^D2/D2 ^N2 and F2 mice, n = 106; *P *= 0.987 for *Bax*^B/B ^vs. *Bax*^D2/B ^and *Bax*^D2/D2 ^F2 mice only, n = 53; and *P *= 0.575 for *Bax*^B/B ^vs. *Bax*^D2/B ^vs. *Bax*^D2/D2 ^F2 mice only, n = 53).

## Discussion

We screened 15 different strains of inbred mice for cell loss caused by optic nerve crush and identified differing levels of susceptibility. DBA/2J mice were identified as the most resistant strain, and BALB/cByJ mice were identified as the most susceptible strain. Offspring of crosses involving these two lines inherited the resistance phenotype, while a backcross of the F1 mice to the susceptible parental strain, yielded a population of mice with the susceptible phenotype. The progeny of the F1 intercross inherited an intermediate phenotype with a bias toward resistance. The pattern of inheritance exhibited by the crosses suggests involvement of dominant alleles and a Punnett Square analysis to determine the frequency of inheritance of 2 dominant genes, with complete penetrance, accurately predicted the mean cell loss observed in each cross. Similarly, a Wright formula calculation estimated that a minimum of 1.3 loci were affecting this phenotype. Analysis of *Bax *expression level and allelic segregation with phenotype excluded this gene as a candidate for one of the affecting alleles.

### The Influence of Genetic Background on Neurodegeneration in Inbred Mice

Several studies have reported that inbred strains of mice exhibit variable susceptibility to neurodegenerative stimuli, but there has been no consistent correlation between strain and susceptibility. C57BL/6 mice, which were relatively crush resistant in our study, are more susceptible to MPTP exposure, spinal chord injury, and experimental autoimmune encephalitis (EAE) than other strains. Conversely, BALB/c mice (crush susceptible) are resistant to EAE and MPTP, but exhibit a similar susceptibility as C57BL/6 mice to spinal chord injury. C57BL/10 mice (crush resistant) and B10.PL mice, which are EAE resistant and susceptible, respectively, both exhibit less severe injury after spinal chord lesion than other strains [9]. Part of the differences exhibited by some inbred strains may reside in their variable abilities to mount an immune response, which can affect the progression and severity of certain neurodegenerative diseases [9,31]. The lack of a correlation between mouse strains and their susceptibility to different degenerative challenges is not surprising. Each challenge is potentially unique to different subsets of neurons (such as cells in the substantia nigra being susceptible to MPTP). It is also likely that different diseases elicit different damaging stimuli, which in turn can activate alternative pathways leading to neuronal death [24,32].

### Genes that affect Retinal Ganglion Cell Death

Recently, Libby et al [14] outlined two broad classes of genes that can affect ganglion cell susceptibility to damage in glaucoma. The first class modulates extrinsic susceptibility of ganglion cells. This set of genes is predicted to influence the response of non-ganglion cells (e.g., glia) to ocular hypertension, the composition and modulation of the extracellular matrix, the density and regulation of retinal vasculature of the optic nerve head, and possibly the immune responses in the eye. The second class of genes modulates the intrinsic susceptibility of ganglion cells. These latter genes are predicted to directly affect ganglion cell survival. By the nature of our screen, which directly activates apoptosis by axonal lesion, we predict that we are most likely selecting for intrinsic susceptibility genes.

Many genes could be intrinsic susceptibility factors. Retinal ganglion cell death follows the basic molecular pathways identified in other neurons and several of the key genes involved in cell death could affect ganglion cell susceptibility to damaging stimuli. We examined the *Bax *gene as a candidate based on the evidence that this gene was critical for ganglion cell death in a variety of situations [23-25]. Several other genes, however, are also known to influence ganglion cell death. Proapoptotic genes active in ganglion cells include *c-Jun *[33,34] and c-JUN terminal Kinase (*Jnk*) [35], *p53 *[36], *Bim *[37], *Apaf-1 *[34], poly(ADP-ribose) polymerase (*Parp*) [38], and several caspases [39-42]. Interestingly, a significant association has been reported between p53 (TP53) haplotypes and primary open angle glaucoma in humans [43].

In addition to proapoptotic genes, ganglion cells are also significantly affected by the expression of antiapoptotic genes. Ganglion cells express *BclX*, whose expression decreases during the initial stages of apoptosis [44]. Upregulation of this gene in ganglion cells enhances their survival [45]. Similarly, transgenic mice that overexpress the *BclX *homolog, *Bcl2*, exhibit nearly complete abrogation of ganglion cell death during development and after optic nerve transection [46,47]. Since downregulation of endogenous *BclX*, along with other normally expressed genes [48,49], is an early event in ganglion cell apoptosis, any variation that reduces this process and allows these proteins to persist could affect the normal apoptotic program.

Damaged ganglion cells in mammals also exhibit an early stress response that includes the up-regulation of heat shock proteins [50,51] and iron-regulating genes including transferrin and ceruloplasmin [52,53]. These latter genes may also act as antioxidants by scavenging free radicals formed from mitochondria during the cell death process [54].

Although *Bax *has been eliminated as a candidate gene in this population of mice, clearly there are other genes that could affect the cell death phenotype. We are currently in the process of conducting a genome wide screen to identify potential loci that affect the resistance phenotype.

### Using Mice to Identify Susceptibility Alleles for Glaucoma

In this study, we used inbred mice to assess if genetic background affects the susceptibility of retinal ganglion cells to a lesion of the optic nerve. These experiments do not directly address whether the same genes that affect ganglion cells after optic nerve crush will also be susceptibility alleles for ganglion cells exposed to elevated levels of IOP, the principal damaging insult in glaucoma. The actual mechanism linking high IOP with ganglion cell damage is still unresolved, but some studies suggest that elevated pressure causes increased sheer forces on the optic nerve at the level of the scleral canal and lamina cribrosa [55,56]. This has lead to the hypothesis that increased IOP causes mechanical damage to axons at this region [57,58], and it is becoming more evident that this is the region of initial damage in glaucoma [59-61]. Although the concept of sheer stress acting directly on the axons is attractive, and could be analogous to our crush insult, there are several other mechanisms by which IOP can induce damage in this region. These include reducing the perfusion of microvessels in the nerve head and the focal activation of astrocytes and microglia. Consequently, it is possible that optic nerve crush may act differently on ganglion cells than elevated IOP.

Regardless of the nature of the insult, there is some evidence, however, both optic nerve crush and elevated IOP result in a similar cell death response within the ganglion cells themselves. Ganglion cell apoptosis is *Bax*-dependent [24,25] and leads to the activation of caspases [39,41,42] in both conditions. In addition, ganglion cells undergo early losses of gene expression before the activation of detectable ganglion cell loss [48] after crush and in glaucoma. In view of the similarities of the cell death pathways activated by both conditions, we predict that the susceptibility alleles that affect ganglion cell death after crush will also affect ganglion cell loss in glaucoma. A critical test of this hypothesis is to first identify the loci that affect the crush phenotype and then assess how the same gene(s) affect ganglion cell loss in glaucoma. For example, DBA/2J mice naturally develop a form of secondary chronic inherited glaucoma [62-65]. Although DBA/2J mice are the most resistant strain assayed in our study, they still exhibit variable susceptibility to elevated IOP as they age, which eventually leads to ganglion cell loss. Breeding the susceptibility alleles inherent in BALB/cByJ mice onto the DBA/2J background, however, could result in mice that develop a more aggressive form of glaucoma. Thus, in response to the same levels of elevated IOP, these modified mice may develop earlier onset of ganglion cell loss or perhaps an accelerated rate of retinal degeneration.

## Conclusion

In summary, our examination of ganglion cell loss after optic nerve crush in inbred mice indicates that genetic background influences the susceptibility of these cells. Experiments using a reciprocal breeding strategy demonstrate that the resistance phenotype is inherited in a dominant fashion and involves relatively few loci. Extending this observation to identify the genes at these loci, may help elucidate some of the alleles that contribute to susceptibility to ocular hypertension leading to glaucoma in humans.

## Methods

### Inbred mice

Animals were handled in accordance with the Association for Research in Vision and Ophthalmology Statement on the use of animals for research. All inbred mice were purchased from the Jackson Laboratory (Bar Harbor, ME). Five female and 5 male mice, aged 6 weeks, were obtained from each of the following lines: 129X1/2J, A/J, BALB/cByJ, C3H/HeJ, C57BL/6J, C57BL/10J, CBA/CaJ, DBA/1J, DBA/2J, FVB/NJ, LG/J, MRL/MpJ, NOD/LtJ, NZB/BINJ, SJL/J. Lines were chosen to obtain a cross section of mice with different coat colors, varying degrees of neurosensory defects (including 8 lines with hearing loss due to homozygosity for the *Cdh23*^*ahl *^allele and 3 lines with photoreceptor degeneration due to homozygosity for the *Pde6b*^*rd1 *^allele), and at least 2 pairs of lines with similar genetic backgrounds (C57BL/6J – C57BL/10J and DBA/1J – DBA/2J). DBA/2J mice also exhibit spontaneous chronic glaucoma associated with the loss of ganglion cells [62,65]. This disease affects older mice and was not expected to influence the response of young mice to an acute lesion.

Mice were aged to 8 weeks, at which time they underwent optic nerve crush on the left eye (see below). Mice were allowed to recover and were maintained for another 2 weeks before being euthanized for cell counting. This protocol was strictly adhered to for all the mice examined in this study. New animals were obtained from the Jackson Laboratory for all matings that required the original inbred parental strains. A reciprocal breeding strategy was used for subsequent matings to generate N2 backcrossed mice (F1 × susceptible parental strain) and F2 mice (F1 intercross).

### Optic nerve crush and ganglion cell counting

For optic nerve crush surgery, mice were anesthetized with ketamine (6 mg/mL) and xylazine (0.4 mg/mL). The crush protocol was performed as described previously [26] using an intraorbital approach. Only the left eye of each mouse underwent the procedure. The loss of ganglion cells is a continuous process over a 3–4 week period, therefore we examined the time point at which there was the earliest maximum difference between the resistant and susceptible strains. Both DBA/2J and BALB/cByJ mice exhibit minimal cell loss at one week, followed by a period of rapid cell loss between 1 and 2 weeks. Each strain also exhibits modest cell loss after this point, but the difference between the two is not different than that observed at 2 weeks after crush (data not shown). For this reason, we examined all populations of mice at 2 weeks after optic nerve crush.

To quantify cell loss, mice were euthanized and the superior region of each eye was marked with an ophthalmic cautery prior to enucleation. Each eye was then enucleated and fixed for 1 h at 22°C in 4% paraformaldehyde in 0.1 M phosphate buffer (pH 7.4) containing 100 mM NaCl (PBS) followed by rinsing in PBS. An eyecup of the posterior pole was isolated and incubated in PBS containing 0.3% TritonX-100 (v/v), overnight at 22°C. Following incubation, the retina was dissected and mounted (ganglion cell layer up) on a glass "Plus" slide (Fisher Scientific, Chicago, IL), dried, and flattened under a coverslip weighted with a 10 g weight [66]. Dry retinas were stained by painting them with 1% cresyl violet acetate in 0.25% acetic acid [67]. Stained retinas were then differentiated and dehydrated in 100% ethanol, cleared in xylene, and coverslipped. Each slide contained the control and experimental retinas from a single mouse.

For counting cells in the ganglion cell layer, we captured digital images taken at 200× magnification using an Olympus BX40 light microscope (Olympus, Mellville, NY) with a SONY DXC-390 video camera attachment (Sony, New York, NY) and imported them into Image Pro Plus v4.5 (Media Cybernetics, Inc., Silver Springs, MD) quantification software. Parameters were set to automatically identify cells with round nuclei greater than 4 μm in diameter and exclude vascular endothelial cells (with spindle-shaped nuclei) and small densely staining cells that are common in the mouse retina [68]. The selection process did not distinguish between ganglion cells and large amacrine cells, but previous studies have estimated the proportion of ganglion cells in this layer to be 40–60% of the neurons present [26,68,69]. The number of cells present in a minimum of 4 microscopic fields, each encompassing an area of 0.33 mm^2 ^and taken in the peripheral to midperipheral region around the 4 quadrants of each retina, was counted. In non-experimental retinas, the total numbers of cells ranged from 2055 – 2913 per field, consistent with previous reports of neuronal cell density in this layer [69]. The numbers of cells present in each retina was taken as the average of these counts and cell loss was calculated in each mouse as the percent change between the control and experimental eyes. Overall, we counted approximately 10% of the neurons present in each retina, which previous studies have demonstrated provides an accurate estimate of the loss of retinal ganglion cells after optic nerve crush [25,26,48].

Ganglion cell number was also estimated in untreated eyes of DBA/2J and BALB/cByJ mice by axon counting. Briefly, the optic nerves of 5 eyes from each strain were harvested and fixed overnight in 2.5% glutaraldehyde in PBS and then embedded in glycol methacrylate (JB-4 Plus, PolyScience, Worthington, PA). Transverse sections of 2 μm thick were cut approximately 1 mm from the globe and stained by silver impregnation to identify axons [67]. Axon counting was conducted as described previously [36] using 3 sections from each nerve.

### Ribonuclease protection assays (RPAs)

Total RNA was isolated from the brains of DBA/2J (n = 24) or BALB/cByJ (n = 23) mice. Radiolabeled antisense riboprobes for RPA were synthesized from linearized pBK-CMV plasmid (Stratagene, La Jolla, CA) templates containing the terminal 127 nucleotides of the murine *Bax *coding region or nucleotides 55–252 of the murine *S16 *ribosomal protein coding region. Probe synthesis, purification, hybridization, and RNase digestion conditions were as described previously [48]. Because retinas were used for the quantification of cell loss, RPA assays were performed on total RNA isolated from brain tissue of mice. In some mice, however, we confirmed that the level of *Bax *mRNA, and the ratio of *Bax *mRNA to *S16 *mRNA, was consistent between the 2 CNS tissues (data not shown). RPAs were initiated by precipitating 20 μg brain total RNA for *Bax *or 1 μg brain total RNA for *S16 *with 5 × 10^5 ^acid precipitable cpm of probe. After hybridization and RNase digestion, protected probes were separated on 0.4 mm 8 M urea/6% polyacrylamide gels, and exposed to BioMax MR film (Kodak, Rochester, NY). Exposure times ranged from overnight to 10 d at -80°C with an intensifying screen. Band intensities were quantified from densitometric scans of multiple exposures of each gel using NIH Image software, and confirmed by liquid scintillation counting of the excised bands.

### Genotyping mice

Genomic DNA was isolated from the spleens of N2 backcrossed mice (n = 55) and F2 mice (n = 56) for genotyping. *Bax *is located at position 23.0 on chromosome 7. PCR genotyping analysis was performed for the polymorphic marker D7Mit27 using the primers: 5' TGA ACT GGG GAG GAA AGT TG and 5' AAC ATG AAA AGA CAT TCC CCC. These primers yielded a 218 bp fragment for DBA/2J mice and a 182 bp fragment for BALB/cByJ mice and map within 1.5 Mbp of the mouse *Bax *gene. PCR products were separated on 3% Meta Phor Agarose (Cambrex, Rockland, ME) gels and the bands were identified by ethidium bromide staining.

## Authors' contributions

RWN (corresponding author) conceived of the study, directed the research, and wrote the manuscript. YL did all the optic nerve crush experiments and phenotyping mice, and made contributions to data analysis and manuscript writing. CLS helped in data analysis and project design and contributed to manuscript preparation. SJS did the *Bax *mRNA quantification and genotyping of mice.

## Figures and Tables

**Table 1:** Matrix of *P *values of the mean cell counts from all inbred lines

Strain	DBA/2J	129X1/SvJ	C57BL/10J	DBA/1J	C57BL/6J	MRL/MpJ	A/J	FVB/NJ	CBA/CaJ	LG/J	SJL/J	NZB/BINJ	NOD/LtJ	C3H/HeJ
BALB/cByJ	**0.00012**	**0.00017**	**0.0002**	**0.00001**	**0.0008**	**0.0005**	**0.0008**	**0.0001**	**0.00003**	**0.0004**	**0.01**	**0.015**	**0.003**	0.339
C3H/HeJ	**0.001**	**0.0017**	**0.0022**	**0.0005**	**0.006**	**0.005**	**0.007**	**0.002**	**0.001**	**0.005**	**0.048**	0.066	**0.05**	
NOD/LtJ	**0.001**	**0.004**	**0.004**	**0.0007**	**0.015**	**0.014**	**0.019**	**0.0065**	**0.003**	0.013	0.18	0.29		
NZB/BINJ	**0.0096**	**0.025**	**0.033**	**0.024**	0.069	0.08	0.095	0.077	0.07	0.126	0.35			
SJL/J	**0.022**	0.06	0.07	0.08	0.138	0.17	0.189	0.189	0.194	0.265				
LG/J	**0.04**	0.138	0.162	0.187	0.28	0.344	0.36	0.398	0.42					
CBA/CaJ	**0.034**	0.138	0.166	0.209	0.3	0.386	0.4	0.46						
FVB/NJ	**0.045**	0.165	0.197	0.258	0.336	0.42	0.436							
A/J	0.087	0.239	0.276	0.359	0.407	0.489								
MRL/MpJ	0.079	0.236	0.275	0.365	0.413									
C57BL/6J	0.125	0.322	0.363	0.476										
DBA/1J	0.088	0.308	0.354											
C57BL/10J	0.194	0.458												
129X1/SvJ	0.214													

A table showing head-to-head statistical comparisons of the mean cells remaining of each strain of inbred mice. *P *values (*t*-test) shown in bold font indicate a significant difference in the mean values between the compared mice. Based on the criterion of cell loss in the ganglion cell layer 2 weeks after crush, DBA/2J mice exhibited the greatest resistance to optic nerve crush, while BALB/cByJ mice exhibited the greatest susceptibility.

**Table 2:** Total neuron and ganglion cell counts from DBA/2J and BALB/cByJ mice

Strain	GCL Neurons*	Total Axons**	Percent RGCs
DBA/2J	118416 ± 12313	72175 ± 17554	61.0
BALB/cByJ	90788 ± 11200	54740 ± 8484	60.3

A table showing the comparison of total neurons counted in the ganglion cell layer (GCL) and the percentage of this layer that are made up of retinal ganglion cells (RGC) in DBA/2J and BALB/cByJ mice. RGCs make up a similar percentage of the neurons present in this layer of both DBA/2J and BALB/cByJ mice (61% and 60.3%, respectively), which validates the methodology of quantifying cell loss as a percentage of the total number of neurons present in the GCL of each strain.

*Neurons were identified from Nissl-stained whole mounts of untreated retinas and counted. A calculation was made of the neuron density/mm^2 ^from a minimum of four 200× microscopic fields of at least 30 non-experimental retinas per strain. The total number of neurons was then calculated by multiplying the density by the total retinal area measured in the whole mounts using Image Pro software.

**Axon counts were generated from a minimum of three 1000× fields per section (3 sections per nerve) of 5 individual optic nerves per strain. The density of the axons was then calculated and total axon number was determined by multiplying the density by the total nerve area measured in the section using Image Pro software.
